# Use of Functional MRI in Deep Brain Stimulation in Parkinson's Diseases: A Systematic Review

**DOI:** 10.3389/fneur.2022.849918

**Published:** 2022-03-23

**Authors:** Jingya Miao, Mohamed Tantawi, Victoria Koa, Ashley B. Zhang, Veronica Zhang, Ashwini Sharan, Chengyuan Wu, Caio M. Matias

**Affiliations:** ^1^Department of Neurosurgery, Thomas Jefferson University, Philadelphia, PA, United States; ^2^Jefferson Integrated Magnetic Resonance Imaging Center, Department of Radiology, Thomas Jefferson University, Philadelphia, PA, United States

**Keywords:** Parkinson's disease, functional connectivity, DBS, deep brain stimulation, fMRI, functional MRI, neuroimaging

## Abstract

Deep brain stimulation (DBS) has been used to modulate aberrant circuits associated with Parkinson's disease (PD) for decades and has shown robust therapeutic benefits. However, the mechanism of action of DBS remains incompletely understood. With technological advances, there is an emerging use of functional magnetic resonance imaging (fMRI) after DBS implantation to explore the effects of stimulation on brain networks in PD. This systematic review was designed following the Preferred Reporting Items for Systematic Reviews and Meta-Analyses (PRISMA) guidelines to summarize peer-reviewed articles published within the past 10 years in which fMRI was employed on patients with PD-DBS. Search in PubMed database provided 353 references, and screenings resulted in a total of 19 studies for qualitative synthesis regarding study designs (fMRI scan timepoints and paradigm), methodology, and PD subtypes. This review concluded that fMRI may be used in patients with PD-DBS after proper safety test; resting-state and block-based fMRI designs have been employed to explore the effects of DBS on brain networks and the mechanism of action of the DBS, respectively. With further validation of safety use of fMRI and advances in imaging techniques, fMRI may play an increasingly important role in better understanding of the mechanism of stimulation as well as in improving clinical care to provide subject-specific neuromodulation treatments.

## Introduction

Deep brain stimulation (DBS) is a well-established neurosurgical treatment for Parkinson's disease (PD) that works by modulating aberrant neural circuits *via* electrical stimulation to a key structure, most commonly the subthalamic nucleus (STN) or globus pallidus internus (GPi) (1). DBS has shown both rapid and sustained improvements of PD motor symptoms (1, 2). The effects of DBS on non-motor symptoms have been described, such as PD-related pain (3) and cognitive functions (4), although the efficacy is still controversial (5, 6). Individual PD patients may respond to DBS differently (7) and the underlying therapeutic mechanism of stimulation action remains incompletely understood (1, 2). This is partially due to the complexity of neural circuits, electrical stimulation affecting both locally and globally, the innumerous possible combination of parameters for DBS programming, and the inter-individual variability (1, 2, 8).

Studies have utilized multiple neuroimaging techniques to investigate the modulatory effects of DBS on brain activity, including non-invasive methods, such as positron emission tomography (PET), single-photon emission computed tomography (SPECT), and functional magnetic resonance imaging (fMRI) (8, 9). Compared with PET and SPECT, fMRI provides better spatiotemporal resolution for detecting brain activity across small but distributed areas associated with the basal ganglia (2, 10). Moreover, fMRI can be continuously acquired while DBS is switched on and off (11). It does not require the use of tracers, which introduces confounding variables between subjects due to different metabolic kinetics (8). The challenges of using fMRI in DBS-implanted patients are related to hardware artifact, as well as safety concerns, including the possibilities of lead migration, heating, and DBS hardware malfunction (2, 8); however, both 1.5 and 3 T fMRI scanning have been shown to be feasible and safe with DBS systems both turned OFF and ON (2, 12–14).

This will likely pave the way for additional DBS neuroimaging studies, thereby providing a more comprehensive understanding of the mechanism of DBS and improving clinical care for individual patients with PD. The purpose of this systematic review is to summarize the available literature on the use of fMRI in PD patients who have undergone DBS treatment in terms of important recent findings and the significance of fMRI as a highly informative tool.

## Methods

This systematic review was performed following the Preferred Reporting Items for Systematic Reviews and Meta-Analyses (PRISMA) guidelines (15) to collect scientific studies in which fMRI was employed in PD patients who had undergone DBS implantations. The search was performed in PubMed database to find English-language articles published from January 2010 to May 2021 (last searched date: June 1, 2021) using the combination of keywords: (“function^*^”[All Fields]) AND (“MR”[All Fields] OR “MRI”[All Fields] OR “magnetic resonance”[All Fields]) AND (“DBS”[All Fields] OR “deep brain stimulation”[All Fields]) AND (“parki^*^”[All Fields]). The resulting references were imported to Covidence.org, which automatically removes duplicate articles. Then, the abstracts and titles of the references were screened by two authors for relevance to fMRI in PD-DBS patients. Full text articles were reviewed by five reviewers working independently to screen articles that met the inclusion criteria: human subjects with PD treated with DBS, fMRI acquired after DBS implantation with a purpose relevant to PD. Articles involving patients with PD as controls to study other diseases were not included. Two reviewers resolved possible conflicts to select articles included in this review.

The assessment of study risk of bias was carried out following the Cochrane guidelines (Chapter 8) (16) by evaluating each included article from the following domains: selection, performance, detection, attrition, reporting, and other biases. For each domain, a judgment of high-risk, low-risk, or unclear was determined. Extracted variables from each article were numbers of participants and subjects' states during scanning, fMRI paradigms, timepoints of fMRI acquisition, and analysis methods. Customized table formats were used to group articles and explore possible heterogeneity. Meta-analysis was not performed, as the purpose of this review is to provide qualitative rather than quantitative evaluations.

## Results

### Article Selection

**Figure 1:**
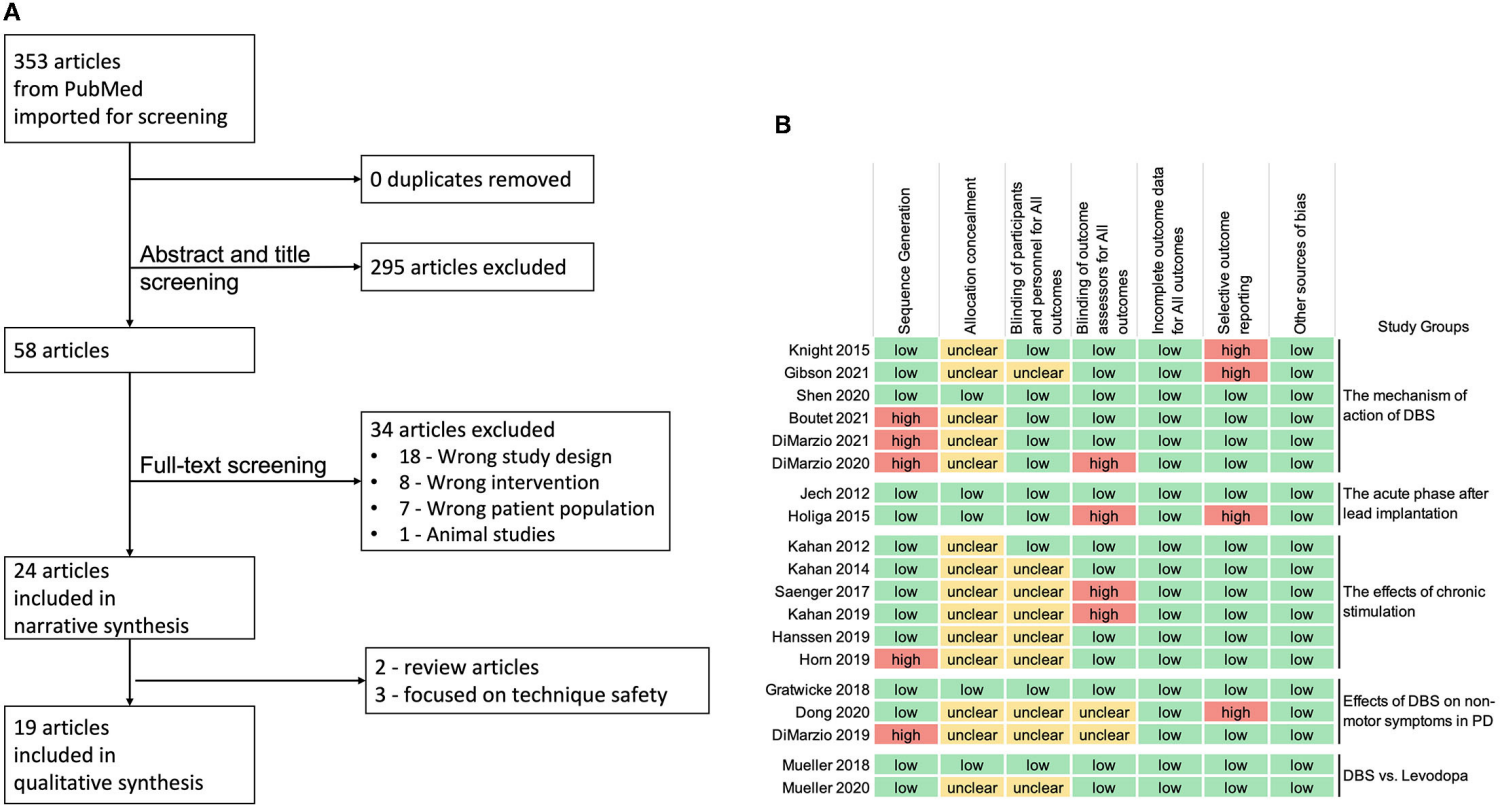
**(A)** Preferred reporting items for systematic reviews and meta-analyses (PRISMA) flowchart. **(B)** Review authors' judgments about each risk of bias domain for the 19 articles included in qualitative synthesis, following the Cochrane guidelines (high-risk in red, low-risk in green, and unclear risk of bias in yellow).

The search strategy described above yielded 353 articles. Following the abstract and title screening, 295 articles were excluded as they were not relevant to the use of fMRI and PD-DBS patients that resulted in 58 articles. Full-text screening excluded 34 articles, which led to 24 articles included for narrative synthesis. Five articles were not comparable due to the type of articles and purposes of their studies, and finally, 19 studies were included for qualitative synthesis (Figure 1).

The assessment of the risk of bias (Figure 1) revealed that 5 studies had high risk of “sequence generation” due to non-counterbalanced scanning conditions of DBS settings (1, 10, 13) or the nature of the study design (17, 18). Most of the studies did not specify if the assessors were blinded (allocation concealment), however, the nature of voxel-based fMRI imaging analysis (such as, preprocessing and FDR corrected *p*-values lowered the risk of bias.

### Study Characteristics

The fMRI design paradigms that were used in the included articles were categorized into three groups: (1) resting-state fMRI (rs-fMRI), during which subjects were asked to remain relaxed for 6–10 min (2, 4, 7, 10, 14, 19–26). (2) A DBS ON/OFF block design, wherein stimulation was cycled ON and OFF while the subject laid still in the scanner, to investigate the mechanism of action of DBS (1, 2, 11, 13, 18, 27). (3) Behavior-dependent task-based design, where a behavioral or stimulus task was interleaved with rest while DBS was either ON or OFF during each session (9, 17, 22, 28, 29) (Table 1). The main approaches of fMRI data analysis used in the reviewed studies included functional connectivity (FC), effective connectivity (EC), eigenvector centrality mapping (ECM), and contrast images (Table 1). In addition, these metrics were correlated with clinical measurements, such as UPDRS-III score, and/or imported to machine learning models. The 19 studies included for qualitative synthesis were grouped according to study purposes and fMRI paradigms: the mechanism of action of DBS, the acute phase after lead implantation, the effects of chronic stimulation, the effects of DBS on non-motor symptoms in PD, and DBS vs. Levodopa (Table 2).

**Table 1:** Summary of the types of functional MRI (fMRI) paradigms and analyses.

**fMRI paradigm**	**Descriptions**
Resting-state fMRI	Subjects remain relaxed for 6–10 mins, during which the DBS was either ON or OFF
DBS ON/OFF block design	Subjects laid still while the DBS was switched ON and OFF for 30 s in each state. This is to mimic the conventional task-based fMRI paradigm
Behavior-dependent task-based design	Subjects were asked to perform a task (or receive stimulus), while DBS was either ON or OFF during the scan session
**fMRI analysis**	**Descriptions**
Functional connectivity (FC)	A statistical correlation of brain activity indicating the synchronization between regions and/or voxels
Effective connectivity (EC)	The directional influence that a brain region has over another region indicating a causal relationship between these two regions
Eigenvector centrality mapping (ECM)	A data-driven and parameter-free analysis technique based on graph theory, which can detect central hubs that are strongly connected to a brain network
Contrast images	Differences in brain activation during task/DBS-ON compared to that during baseline/DBS-OFF

**Table 2:** Summary of included articles for qualitative analyses.

**References**	**Subj # PD (HC)**	**Target**	**MRI scanner**	**fMRI category**	**Major fMRI measurements timepoints and scanning conditions**		Notes ^*^
**The mechanism of action of DBS**							
Knight et al. (11)	10	STN	1.5 T	DBS ON/OFF block	0-3 days post-op	· DBS ON/OFF cycling (6 s ON and 60 s OFF)	
						· 2 V, 90 microsecond, 130–180 Hz	
						· Awake or under general anesthesia	
Gibson et al. (27)	20	STN	1.5 T	DBS ON/OFF block	0–3 days post-op	· DBS ON/OFF cycling (6 s ON and 60 s OFF)	
						· 3 V, 90 ms, 130 Hz	
						· Under general anesthesia	
Shen et al. (2)	14	STN	3 T	DBS ON/OFF block	1, 3, 6, 12 mos post-op	· DBS ON/OFF cycling (36 s ON and 24 s OFF)	Bilateral stimulation
						· Stimulation with low (60 Hz) or high (120 Hz) frequency	
						· 60 min wash-out between fMRI sessions	
Boutet et al. (1)	39	STN GPi (*n* = 4)	3 T	DBS ON/OFF block	Mean 20.5 mos post-op	· DBS 30 s ON/OFF cycling	
						· Left stimulation with optimal, followed by non-optimal contact or voltage ^**^	
						· Bilateral stimulation with low or high frequency	
						· 15 min wash-out time only before the first fMRI scan	
Dimarzio et al. (13)	14	STN	1.5 T and 3 T	DBS ON/OFF block	Post-op after DBS optimized	· DBS 30 s ON/OFF cycling	Some subjects were scanned with meds-ON
						· Medication doses continued	
						· Stimulation with optimal settings (mono- or bipolar-)	
						· Followed by altered frequency by ± 30 Hz, ± 60 Hz relative to individual's optimal frequency ^**^	
						· <5 min between fMRI sessions	
DiMarzio et al. (18)	23	STN GPi (*n* = 8)	1.5T and 3T	DBS ON/OFF block	Post-op after DBS optimized	· DBS 30 s ON/OFF cycling	Subjects were scanned with meds-ON
						· Medication doses continued	
						· DBS with clinically optimal settings	
						· 5 min wash-in time before ON/OFF cycling session	
**The acute phase after lead implantation**							
Jech et al. (9)	12	STN	1.5T	Finger-tapping task	Pre-op	· Med-off and DBS-OFF	
					0–3 days post-op		
Holiga et al. (21)	13	STN	1.5T	Resting state	Pre-op	· Med-off, DBS ON and OFF	
					0–3 days post-op		
						· Unilateral bipolar stimulation	
						· 2.64 ± 0.44 V, 60 microsecond, 130 Hz	
**The effects of chronic stimulation**							
Kahan et al. (28)	10	STN	1.5 T	Joystick-motion task	>6 mos post-op	· Med-off, DBS-ON and OFF	
Kahan et al. (14)	12	STN	1.5 T	Resting state	>6 mos post-op	· Med-off, DBS-ON and OFF	
Saenger et al. (24)	10 (56)	STN	1.5 T	Resting state	>6 mos post-op	· Med-off, DBS-ON and OFF	
Kahan et al. (22)	11	STN	1.5 T	Resting state	>3 mos post-op	· Med-off, DBS-ON and OFF	
				Joystick-motion task		· Med-off, DBS-ON and OFF	
Hanssen et al. (20)	26	STN	1.5 T	Resting state	3–78 mos post-op	· Med-on, DBS-ON and OFF	
Horn et al. (10)	20 (15)	STN	1.5 T	Resting state	>4 mos post-op	· Med-on, DBS-ON followed by DBS-OFF^**^	
						· 5–15min wash-out time until symptoms reappeared	
**Effects of DBS on non-motor symptoms in PD**							
Gratwicke et al. (19)	6	NBM	1.5 T	Resting state	Post-op (after 6 weeks of DBS/sham)	· 2-week washout period	Symptom: dementia
Dong et al. (4)	23(14)	STN	1.5 T	Resting state	Pre-op	· Med-off, DBS-OFF	Symptom: executive functions
					>3 mos post-op		
Dimarzio et al. (17)	15	STN	1.5 T and 3 T	Pain-stimulus task	Post-op	· Med-off, DBS-ON and OFF	Symptom: chronic pain
**DBS vs. Levodopa**							
Mueller et al. (23)	13	STN	1.5 T	Resting state	Pre-op	· Med-on and off	
					0–3 days post-op	· Med-off, DBS-ON and OFF	
Mueller et al. (29)	32	STN	1.5 T	Finger-tapping task	Pre-op	· Med-on and off	
	18				0–3 days post-op	· Med-off, DBS ON and OFF	

* *Intraoperative timepoint is post lead implantation and before the implantation of stimulator. All of the post-operative fMRI acquisition was performed while the subjects were OFF medication, unless specified. All of the deep brain stimulation (DBS) stimulation settings during DBS-ON fMRI scan was unilateral bipolar stimulation, unless specified*.

** *Order of fMRI sessions were not counterbalanced*.

*STN, subthalamic nucleus; GPi, globus pallidus internus; NBM, nucleus basalis of Meynert*.

### The Mechanism of Action of DBS

Six original studies employed the DBS ON/OFF block paradigm in their fMRI scans to investigate the immediate changes in BOLD signals induced by stimulation ON vs. OFF at various post-operative timepoints ranging from the same day of patients' lead implantation surgery (11, 27) to over 2-year post-implantation (1, 2, 13, 18). Nearly all recruited patients had DBS implanted in the STN, except that two studies also included a number of GPi DBS patients and analyzed both targets as a single group (1, 18).

Contrast images of DBS ON-OFF revealed some similar neurocircuit responses across independent cohorts regardless of ON/OFF block length or duration post-operation: (a) significant activation of thalamus was observed in all of six studies, (b) significant activation of pallidum in three studies (2, 13, 18), (c) significant deactivation of cerebellum in two studies (1, 2), while increased cerebellar activity found to be associated with side effects (27), and (d) significant changes of the primary motor cortex (M1) of which increased activation seen in rigidity phenotypes (18, 27), whereas significant deactivation seen in tremor-dominant phenotypes (1, 2, 13, 18). Of these regions of interests (ROIs), two circuits were identified showing opposite responses to DBS: the activated GPi-circuit covering the GPi, thalamus, and deep cerebellar nuclei and the deactivated M1-circuit covering the primary motor cortex, putamen, and cerebellum. They were found functionally dissociable based on the pre-operative resting state FC analysis (2).

Moreover, BOLD responses to STN DBS were demonstrated to be correlated with motor symptom subscales and specific clinical outcomes (2, 18, 27). The rigidity subgroup responded with activated M1 and SMA (18), and its improvements were significantly correlated with the higher activation in M1 (27) and the GPi-circuit (2). The tremor-dominant subgroup responded with deactivated M1 (18), and its improvements were associated with the higher activation in thalamus (27). Activation in the cerebellar and sensorimotor cortices were correlated with paresthesia and nausea side effects; and activation in the caudate and putamen regions were correlated with dystonia side effects (27). In comparison with STN stimulation, GPi DBS induced BOLD responses were similar in the rigidity and tremor subgroups; however, in the subgroup with postural instability gait disorder, GPi DBS induced M1 deactivation whereas STN stimulation resulted in the M1 activation and better clinical improvement (18).

Using the same fMRI paradigm, three studies explored how stimulation parameters (i.e., contact, voltage, or frequency) affect the functional activity by assessing stimulation-induced BOLD responses obtained under clinically optimized settings, followed by non-optimized settings during which only one parameter was altered (1, 2, 13). Compared with the fMRI signatures of the optimal DBS settings (activation in the thalamus and deactivation in M1 and anterior cerebellum), stimulation at non-optimal contacts led to a diminished magnitude in M1 and increased signals in non-motor cortex (1). Lower-voltage stimulation did not change the topographic pattern but reduced the magnitude of BOLD signals; while high-voltage stimulation produced stronger BOLD signals but accompanied by increased activation in non-motor regions (1). The frequency parameter significantly affected the GPi-thalamus-cerebellum circuit, but not the M1-putamen-cerebellum circuit (2). The optimal stimulation frequency induced the strongest activation in the GPi-circuit, while slightly increased frequency (+30 Hz) resulted in decreased activation in primary somatosensory cortex (13). Another important modulatory factor of DBS treatment is time. Deactivation of the M1-putamen-cerebellum circuit gradually increased over time within 12 months post-surgery (2). This issue of timing of the postoperative MRI scan may be able to explain the inconsistent findings of brain activity in M1 (1, 2, 11, 27).

### The Acute Phase After Lead Implantation

Two studies focused on changes in brain activation associated with the microlesion effect (MLE)—a phenomenon where electrode implantation into the STN or GPi is associated with motor improvement prior to the onset of stimulation (9, 21, 30, 31). The mechanism behind MLE was assessed by fMRI scanned with DBS-OFF 0–3 days after implantation compared with pre-operative fMRI data (9, 21). In movement state, *via* finger tapping task-based fMRI, the amplitude of BOLD responses was found significantly decreased in the motor cortex, insula, thalamus, and basal ganglia, after edema scores were considered as covariates. Besides, the improvements of rigidity and axial UPDRS-III scores were significantly correlated with the BOLD signals in the putamen and globus pallidus (9). In resting state, a data-driven ECM analysis of the whole brain revealed that the brainstem (specifically 2 clusters in the upper and lower brainstem) acted as a compensatory hub in the motor network to likely counterbalance the physical disruption from electrode penetration and microlesion. The EC in the brainstem hubs were inversely correlated with the sub-scores of the UPDRS-III, regardless of surgery stage (combined pre- and post-operative DBS-OFF UPDRS-III scores). After DBS was activated, EC in the left premotor cortex increased, and FC analysis seeded in the brainstem hubs showed significant increased connectivity with the cerebellum (21). These fMRI study findings indicated that microlesion affected BOLD responses to stimulation with a compensating activation in the brainstem, which is different from the mechanism of action DBS described in previous section, even though UPDRS-III scores obtained during and after the microlesion effect were similar.

### Effects of Chronic Stimulation on Motor Networks

Six studies investigated the effects of chronic stimulation therapy in patients who had received STN DBS treatment for at least 3 months, and conducted fMRI, during which each patient was at rest with DBS either ON or OFF (10, 14, 20, 22, 24) or was performing a voluntary movement task (22, 28). The order of DBS ON/OFF was counterbalanced in five studies with no mentioning of the washout time before fMRI with DBS OFF (14, 20, 22, 24, 28); one study acquired fMRI with DBS-ON first, then turned off the stimulation and waited until symptoms reappeared before scanning with DBS-OFF (10).

Various analyses approaches were used. Horn et al. assessed voxel-wise FC of the motor network within the basal ganglia-cerebellar-cortical circuit in two different scenarios: DBS ON vs. DBS OFF. There was increased average connectivity within motor network during DBS ON vs. OFF, specifically by increasing the FC between thalamus and motor cortex while reducing the coupling between striatal and three regions, namely, GPe, STN, and cerebellum. Moreover, the amount to which average connectivity increased was found to be associated with the volume of activated motor STN (10). Kahan et al. constructed a number of hypothetical neural architectures using the dynamic causal models (DCMs) from 5 ROIs (M1, putamen, thalamus, cerebellum, and STN). The DCM of best fit indicated that, at resting state, DBS mostly affect the cortico-basal ganglia circuit by increasing the coupling strengths of M1-putamen, thalamo-M1, and putamen-thalamus pathway and significantly reducing the connectivity of M1-STN, putamen-STN, and STN-thalamus pathways, with no impacts on cerebellar connectivity (22). Another study by Hanssen et al. used a similar approach but with 7 ROIs (M1, SMA, PMC, PFC, putamen, thalamus, and cerebellum). Cerebellar effective connectivity was significantly increased comparing DBS ON vs. OFF, specifically the cerebello-putamen and prefronto-cerebellar circuits. Additionally, the resting tremor improvement was found to be correlated with DBS-induced increased prefronto-cerebellar interaction (20). Kahan et al. performed the same DCM analysis on task-based fMRI data during voluntary movement from the same cohort and revealed a different architecture model with an additional recruitment of cerebellar-basal ganglia interactions. Active stimulation resulted in an increased effective connectivity of STN afferent (M1-STN and putamen-STN) pathways during voluntary movement, whereas a reduced coupling strength during resting state (22).

Deep brain stimulation (DBS) modulatory effects were observed in both behavior independent and dependent statuses, but different inter-regional connectivity was affected: when subjects were at rest, basal ganglia pathways were modulated without the inclusion of cerebellum, while subcortical-cerebellar pathways were activated when subjects were performing voluntary movement (22). However, two other studies on resting state fMRI revealed inconsistent findings, in which cerebellar connectivities were also modulated by therapeutic DBS, but in the opposite directions, with one study representing increased (20) while the other study showing decreased striatal-cerebellum connectivity (10).

Two of the six studies recruited age-matched healthy control subjects for comparisons with each DBS-ON and DBS-OFF conditions, and demonstrated that therapeutic DBS helps in rebalancing resting state brain activities toward healthy controls on a local as well as global level (10, 24). Regarding static FC, FC maps were estimated by seeding from the activated motor STN to the rest of the brain from rs-fMRI acquired with DBS ON and OFF conditions, and a healthy age-matched control group (10). The similarity of FC maps, compared using spatial correlation values, were significantly higher between DBS-ON and healthy controls vs. DBS-OFF and healthy controls (10). Similarly, in terms of dynamic FC, which is used to describe the oscillation of inter-region synchronization throughout the scan time, therapeutic stimulation was found to increase “phase consistency” (defined as the mean and standard deviation (SD) of all windowed FC matrices of individual subject) toward the ones obtained from age-matched healthy controls (24).

### Effects of DBS on Non-motor Symptoms in PD

Three of the reviewed studies looked into the effects of DBS with regards to non-motor symptoms in PD (e.g., chronic pain, dementia, and executive function) using rs-fMRI (4, 19) or block-design task-based fMRI (4). DiMarzio et al. investigated how STN stimulation affects pain perception in PD-DBS patients with chronic pain relief contrasted with those without pain relief. Brain activation corresponding to pain perception was measured using a task-based fMRI paradigm, during which mechanical pain stimulus alternated with resting periods while the DBS was ON or OFF throughout the scan time. Distinct patterns of brain activation were observed: PD patients with pain relief responded to pain with hyperactivation in primary sensory cortex (S1) and anterior cingulate cortex (ACC) during DBS OFF, and turning on the stimulation reduced such activation in these two regions; conversely, PD patients without pain relief showed deactivation in S1 and ACC during DBS OFF, and stimulation ON increased the activation in the two regions (17).

Gratwicke et al. recruited PD patients with dementia and conducted two rs-fMRI scans on each subject after receiving 6 weeks of sham and DBS treatment in the Nucleus Basalis of Meynert (NBM), received in counterbalanced order with 2 weeks of washout intervals. FC in the default mode network revealed no significant differences between the NBM DBS and sham treatments, agreeing with their clinical measurements (19). Dong et al. studied the executive functions in PD patients, who received at least 3 months of STN-DBS, by assessing the intrinsic FC of the executive control network from pre- and post-operative rs-fMRI acquired in DBS-OFF condition. Although both pre- and post-operative rs-fMRI revealed significantly decreased FC comparing with a healthy control group, no significant differences were found between pre- and post-states (4).

### DBS vs. Levodopa

Mueller et al. compared the effects of oral levodopa (L-DOPA) and STN-DBS in individual patients with PD (i.e., within-subject comparison) using the scores of UPDRS-III, rs-fMRI, and finger tapping task-based fMRI—all collected in four scenarios: pre-operative L-DOPA-OFF, pre-operative L-DOPA-ON, post-operative L-DOPA-OFF and DBS-ON, and post-operative L-DOPA-OFF and DBS-OFF. The UPDRS-III scores showed comparable improvements by L-DOPA and DBS from the baseline of the pre-op L-DOPA-OFF scores (23, 29). However, rs-fMRI data revealed different motor network connectivity modulations caused by these two interventions, specifically, DBS-ON increased EC in the bilateral motor cortices accompanied with the increased connectivity with the thalamus and cerebellum compared with L-DOPA-ON (23). In a later study, finger tapping task-based fMRI was assessed *via* the same study design, and beta images during TAPPING and REST were computed for each scan scenario. It was found that L-DOPA-ON reverted putamen activation to increased activation during TAPPING-REST, whereas these reversed patterns of putamen and motor cortex were not found in DBS-ON vs. OFF scenario (29).

## Discussion

This systematic review focused on summarizing the findings of articles published within the past 10 years in which fMRI was employed on PD-DBS patients. A direct comparison of results is complex due to the significant variability in fMRI design paradigm and connectivity analyses described in the previous section. Performing 1.5 and 3 T fMRI is safe in PD-DBS patients with the use of MRI-compatible DBS (2, 12, 18, 21, 28, 32, 33). The effects of DBS on functional activity and integrity can vary depending on factors, such as duration after the implantation, DBS programming parameters, if the scan is acquired at rest or with movement, PD subtypes, and the conditions of medication intake. Turning the DBS ON produces immediate modulation of the cortico-basal ganglia-thalamo-cortical loop in PD, leading to the increased activation in thalamus and globus pallidum (consistent with the DeLong Model of PD), deactivation in cerebellum, and changes of activation in M1 to correct the motor symptoms (i.e., activation in rigidity whereas deactivation in tremor phenotypes). Furthermore, when compared with age-matched healthy controls, DBS seems to rebalance brain activities at resting state toward healthy subjects. Correlations of BOLD signals with various DBS settings and UPDRS-III subscores demonstrate the advantages of fMRI technique to explore the effects of stimulation between PD subtypes and individuals. The use of fMRI in patients with PD-DBS is gradually growing and will enhance our understandings of the mechanism of DBS in PD with the respect of improving motor and non-motor clinical outcomes. This section addresses how the current data could be used in the clinical setting, such as providing patient-specific surgical planning and identifying the optimal or new targets for various symptoms.

### Safety Concerns and Artifacts

Phantom tests conducted at multiple research centers have shown that patients with DBS implanted may safely undergo 1.5 and 3 T MRI (2, 12, 18, 21, 28, 32, 33). As MRI environment did not interrupt the implanted pulse generator functions, more recent studies used the body-transmit coil for the benefits of better signal-to-noise ratio (22). Although the imaging artifact caused by the DBS device appears as circumferences along the DBS leads and in the frontoparietal cortex area close to DBS wire coils, and although larger artifact is seen in 3 T compared with 1.5 T MRI, it is still limited to the superficial cortex and the signal loss adjacent to the electrode contacts is acceptable (12). Additionally, shorting scan time in 3 T MRI scanner (5.5 min compared with 8 min per scan in 1.5 T MRI scanner) seems to compensate the higher signal-to-noise ratio, and thus pooling fMRI data from 1.5 and 3 T scanners for further analyses becomes feasible (18). Therefore, with a priori safety testing, more recent studies have scanned patients with PD-DBS at their clinically optimal DBS settings, including monopolar stimulation (1, 13, 18).

### The Mechanism of Action of DBS

It has been demonstrated that DBS achieves its clinical effects through modulating not only the local neuronal activity within the target region, but also larger brain networks by propagating along related circuitries (23, 27). However, the exact neuromodulatory mechanism of how active stimulation, and more specifically the changes of stimulation parameters, affect brain networks still remain unclear (1, 2). A fMRI paradigm with DBS ON/OFF cycling was employed by multiple studies in our systematic review to measure immediate BOLD signal changes induced by stimulation ON vs. OFF states (1, 2, 11, 13, 18, 27). This design was validated by a high test-retest reliability at the subject level as well as a high inter-subject consistency within the same group or scanning conditions (2). Variations of study designs existed among the reviewed articles, for example, the block lengths (ON-period ranged from 6 to 30 s, OFF-period ranged from 60 to 30 s), post-operative durations (ranging from the same day of the lead implantation surgery to over 2-year post implantation), wash-in/wash-out durations (e.g., unclearly reported, 5 min wash-in period and 60 min wash-out period), and medication on/off. Washout time following the discontinuation of STN DBS is around 30–50 min, a rapid drop of 0–80% followed by further slow washout, which varies depending on individual disease duration, lead location (34, 35), and patients' maneuver (36). The rapid alternation of stimulation ON and OFF states (ON/OFF cycling) utilized by the studies in this review may not fully capture the entire effects of DBS on functional activation. Future studies should consider employing a longer wash-out period in order to overcome this limitation. Nevertheless, the reviewed studies reached generally consistent findings: STN DBS has significant effects throughout the motor circuitry in PD, preferentially the thalamus, primary motor cortex, pallidum, and cerebellum.

### Effects of DBS on Networks

Although, the non-optimized DBS programming may lead these studies less relevant to conclusions of the therapeutic effects of DBS on neural networks in PD-DBS patients (8), the findings may contain predictive information in the matter of clinical outcomes (27). The current standard-of-care procedure for adjusting DBS parameters is labor-intensive and time-consuming. The complexity of this process has been further compounded by the recent introduction of segmented leads; this increased the possible combinations of parameter configuration (37). Furthermore, the optimization is mostly subjective and dependent on personal and clinical experience rather than objective detailed algorithms to generate personalized DBS settings. For instance, acquisition of fMRI following a programming session could have the potential to demonstrate if activation patterns associated with the improvement in UPDRS-III subscores occurred (such as, increased activation in thalamus and globus pallidum, deactivation in cerebellum, and the changes of activation in M1) with a specific set of parameters. Therefore, neuroimaging biomarkers could assist the efficiency and accuracy in the process of DBS programming for individual patients.

Better understanding of the mechanism of chronic stimulation may provide quantitative neuroimaging evidence for predicting DBS efficacy for individual patients. Of the six studies reviewed in this category, the order of fMRI sessions with DBS ON and OFF were counterbalanced, except for one study by Horn et al. (10), in which 5–15 min of DBS washout was included by waiting for the reappearance of symptoms. Consistent findings across these studies demonstrated the main effects of STN DBS on functional connectome at resting state: stimulation strengthens the couplings of the direct pathway and reduces those of the hyperdirect pathway. However, the results of how STN DBS affects the connectivity between cerebellum and striatum were inconsistent, which might be caused in part due to different conditions of medication intake (on or off). Even when two studies had their subjects continue the medication intake throughout fMRI scans, their results were contradictory (10, 20). Therefore, future studies with consistent fMRI scanning designs are necessary to confirm or further explore the specific connectivity changes between the cerebellum and the basal ganglia following chronic therapeutic DBS.

The complete circuitry involved in non-motor symptoms of PD remains unclear. Nevertheless, symptoms, such as pain are common in patients with PD and affect quality of life significantly (38). It has been shown that up to 80% of PD patients may receive pain relief from STN DBS with different effects depending on the types of PD pain phenotypes (39, 40). Yet, the mechanism behind has yet to be determined. Dimarzio et al. demonstrated the reduction of activation in primary sensory cortex and anterior cingulate cortex after turning DBS ON in patients who experienced pain relief, while the opposite finding in patients without pain relief. Although these findings do not elucidate the entire circuitry involved, the activation status of such areas could potentially be used for patient counseling prior to DBS implantation, e.g., managing expectations regarding pain reduction following DBS (17). The effects of DBS on cognitive functions in PD patients are controversial, with previous studies showing declined, stable, or improved cognitive functions at up to 8-year follow-up; DBS targets (STN vs. GPi) seemed to affect the cognitive outcomes as well (5, 6). The assessments of the resting state FC within the executive control network showed no significant changes in post-operative DBS OFF states from the pre-operative baseline in PD patients who received at least 3 months STN DBS (4). Future studies evaluating the effects of chronic therapeutic DBS on cognition should include both STN and GPi DBS, to provide better insight on the differences between both nuclei.

## Limitations and Future Directions

In this review, we only searched in PubMed database and focused on qualitative synthesis without meta-analysis of the studies. Reviewing of the included articles, fMRI has major advantages in studying PD patients following DBS implantation; however, the scanning and processing methodology of the reviewed studies are not uniform, which limited the generalizability and applicability of the results. Standardized fMRI scanning parameters (e.g., the time period of each block in fMRI DBS ON/OFF block design) and processing pipeline would maximize the benefits of fMRI application in PD patients. Other limitations include safety concerns and susceptibility artifacts which can hinder the proper assessment of FC between brain regions.

Machine learning simulation has emerged as a possible solution. Yan et al. (41) used the deep convolutional generative adversarial networks (DCGAN) model to reconstruct the lost BOLD signals in PD-DBS patients. Not only parts of the imaging data were recovered, but also the machine-learning-model-generated BOLD signals corresponded in time with the original signals. The main advantage of using the DCGAN machine learning model over an oversimplified diffusion model is that the DCGAN is able to reconstruct FC maps specific to individual patients. Further studies are needed to improve the reconstructive accuracy of such models and account for brain shift that occurs during surgery.

The location of DBS electrodes is paramount for clinical improvement in motor function, so identifying the exact location of the electrodes is essential for optimal clinical outcomes (33). The most effective stimulation occurs at places that are most strongly connected to the motor network. Therefore, future research may involve performing fMRI scans on patients pre-operatively with a particular emphasis in identifying contact points that would strongly activate the motor network as this has been shown to result in the best clinical improvement. The ultimate goal would be to develop an artificial intelligence (AI) model that can use clinical data and pre-operative FC maps to accurately identify the best location of the DBS leads and stimulation parameters specific to each patient.

## Conclusions

The recent years have witnessed major advances in fMRI use following the DBS implantation in PD patients. Studies at multiple research centers have provided evidence for performing 1.5 and 3 T fMRI safely in PD-DBS patients with properly designed phantom test and the use of MRI-compatible DBS (2, 12, 18, 32). The effects of DBS on functional activity and integrity have shown to be different depending on a number of factors, namely, the duration after the implantation (microlesion effect), DBS programming parameters, the subject's activity while being scanned (at rest or with movement), PD subtypes, and the conditions of medication intake. fMRI studies with a DBS ON/OFF block paradigm have shown that immediate modulation of the cortico-basal ganglia-thalamo-cortical loop in PD led to significant increased activation in thalamus and globus pallidum (consistent with the DeLong model of PD), deactivation in cerebellum, and changes of activation in M1 to correct the motor symptoms (i.e., activation in rigidity whereas deactivation in tremor phenotypes). Compared with age-matched healthy controls, DBS seems to rebalance brain activities at resting state toward healthy subjects. The findings of significant correlations of BOLD signals with various DBS settings and UPDRS-III subscores further signified the advantages of fMRI technique to explore the effects of stimulation between PD subtypes and individuals. Overall, the use of fMRI in PD-DBS patients is showing a growing attraction to clinicians and researchers, with the aims to enhance our understandings of the mechanism of DBS in PD with the respect of improving motor and non-motor clinical outcomes, providing patient-specific surgical planning, and identifying the optimal or new targets for various symptoms.

## Data Availability Statement

The original contributions presented in the study are included in the article/supplementary material, further inquiries can be directed to the corresponding author.

## Author Contributions

JM did the conceptualization, methodology, screening, evaluation, syntheses, writing of the original draft, and visualization. MT did the screening, evaluation, and writing. VK, AZ, and VZ did the screening and writing. AS did the manuscript review and editing. CW did the methodology and manuscript review and editing. CM did the methodology, manuscript review and editing, and supervision. All authors contributed to the article and approved the submitted version.

## Conflict of Interest

The authors declare that the research was conducted in the absence of any commercial or financial relationships that could be construed as a potential conflict of interest.

## Publisher's Note

All claims expressed in this article are solely those of the authors and do not necessarily represent those of their affiliated organizations, or those of the publisher, the editors and the reviewers. Any product that may be evaluated in this article, or claim that may be made by its manufacturer, is not guaranteed or endorsed by the publisher.
